# When benign mimics malignancy: a visual insight into chondroid lipoma

**DOI:** 10.11604/pamj.2025.52.129.48380

**Published:** 2025-11-26

**Authors:** Suhit Naseri

**Affiliations:** 1 Department of Pathology, Datta Meghe Medical College, Datta Meghe Institute of Higher Education and Research, Wanadongri, Nagpur, Maharashtra, India

**Keywords:** Chondroid lipoma, biphasic soft tissue tumor, t(11, 16)(q13, p13) translocation

## Image in medicine

Chondroid lipoma, a sporadic soft tissue tumor (<0.1% incidence), has admixed adipocytic and chondroid features, creating a diagnostic challenge. Here is a case of a 32-year-old female who presented with a 4cm, painless, mobile right thigh mass for over two years. Ultrasonographic findings of a well-circumscribed, heterogeneous hypoechoic lesion prompted excision due to suspicion of malignancy. The patient underwent local excision, and the postoperative period was uneventful. Histopathology established chondroid lipoma by characteristic biphasic architecture consisting of lipocytic component with mature adipocytes containing unilocular vacuoles and peripheral nuclei. Chondroid component, showing hyaline cartilage-like matrix containing chondrocyte-like lacunae as well as myxoid. Transition areas had nests of lipoblasts blending with chondroid regions within an identifiable lobular architecture. Exclusion was differentially essential, as myxoid liposarcoma usually has an absence of atypical lipoblasts/plexiform vasculature. Whereas, extraskeletal myxoid chondrosarcoma usually lacks fatty infiltration. Diagnosis was solidified via FISH-detected t(11;16)(q13;p13) translocation. This case highlights histopathology's critical role in the diagnosis of chondroid lipoma, specifically in atypical age groups (young adults). The distinctive biphasic morphology, lobularity, and transitional areas of the tumor, with molecular profiling, allow discrimination from aggressive mimics. Surgical excision is definitive therapy with a good prognosis and low recurrence. Accurate diagnosis avoids overtreatment and provides optimal management direction, underlining the importance of the integration of morphological, immunohistochemical, and molecular modalities in the soft-tissue tumor workup.

**Figure 1:**
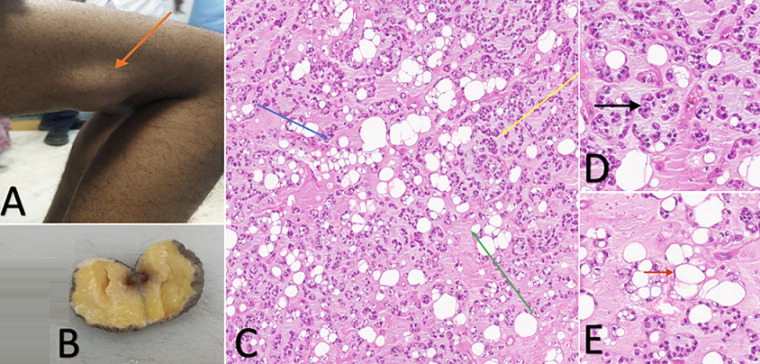
A) lesion on the right thigh (orange arrow); B) cut section of the excised lesion showing encapsulated, yellowish tan, gelatinous lesion; C) H&E, 10x section from the mass showing biphasic architecture- lipocytic component comprising of mature adipocytes containing unilocular vacuoles and peripheral nuclei (blue arrow); chondroid component comprising hyaline cartilage-like matrix containing chondrocyte-like lacunae, as well as myxoid (yellow arrow); transition areas had nests of lipoblasts blending with chondroid regions within an identifiable lobular architecture (green arrow); D) H&E, 40x section showing lipocytic component (black arrow); E) H&E, 40x section showing chondroid component (red arrow)

